# Identification and validation of the dopamine agonist bromocriptine as a novel therapy for high-risk myelodysplastic syndromes and secondary acute myeloid leukemia

**DOI:** 10.18632/oncotarget.6773

**Published:** 2015-12-28

**Authors:** Fabio Giuseppe Liberante, Tara Pouryahya, Mary-Frances McMullin, Shu-Dong Zhang, Kenneth Ian Mills

**Affiliations:** ^1^ Centre for Cancer Research and Cell Biology (CCRCB), Queen's University Belfast, Belfast, United Kingdom

**Keywords:** myelodysplastic syndromes (MDS), acute myeloid leukemia (AML), bromocriptine, re-purposed, therapy

## Abstract

Myelodysplastic syndromes (MDS) represent a broad spectrum of diseases characterized by their clinical manifestation as one or more cytopenias, or a reduction in circulating blood cells. MDS is predominantly a disease of the elderly, with a median age in the UK of around 75. Approximately one third of MDS patients will develop secondary acute myeloid leukemia (sAML) that has a very poor prognosis. Unfortunately, most standard cytotoxic agents are often too toxic for older patients. This means there is a pressing unmet need for novel therapies that have fewer side effects to assist this vulnerable group. This challenge was tackled using bioinformatic analysis of available transcriptomic data to establish a gene-based signature of the development and progression of MDS. This signature was then used to identify novel therapeutic compounds via statistically-significant connectivity mapping. This approach suggested re-purposing an existing and widely-prescribed drug, bromocriptine as a novel potential therapy in these disease settings. This drug has shown selectivity for leukemic cells as well as synergy with current therapies.

## INTRODUCTION

Myelodysplastic syndromes (MDS) are a set of diseases related by their clinical features and underlying etiology and considered a disease of the elderly, with a median age of around 75 [1]. Despite the initial cytopenias caused by dysplastic or failing bone marrow, roughly one third of MDS patients will develop a neoplastic secondary acute myeloid leukemia (sAML) that has a very poor prognosis. Those with high-risk MDS, defined by more severe clinical symptoms and certain cytogenetic features, are most in danger of progression [2]. Unfortunately, most gold-standard chemotherapies elicit excessive toxicity in this relatively frail, older patient group. This has driven research into trying to identify novel therapies, which have fewer side effects, to assist this vulnerable group. Low dose Cytarabine has already been trailed in the elderly population in treating more aggressive myeloid malignancies and successfully reduced disease burden [3, 4]. However, remission and relapse rates are still poor. Traditional drug discovery approaches have been financially and temporally burdensome; relying on either clinical trials in humans or large tissue culture-based drug screening schemes. In recent years, the pharmaceutical world has come to recognize that these methodologies are very inefficient in realizing successful new therapies. This has led to an increase in drug re-purposing strategies that seek to improve the chances of success, by eliminating the pitfalls of first-in-man trials and unpredicted toxicity [5, 6]. One such approach is connectivity mapping (cMAP). First developed by Lamb, et al., it is based on the empirical observation that a transcriptome reflects the state of a cell in a given condition [7, 8]. On this basis, it theorizes that a pattern of transcriptional change induced in one state may be reversed by the opposite pattern of change observed in another state. By example, a cancer cell with a characteristic change in transcription from normal may be phenotypically ‘normalized’ by a drug which triggers an opposite change in the expression of those genes. The two major benefits of this approach are that the initial screen is done *in silico* and that it can allow for re-purposing of existing pharmaceutical agents, as the transcriptional response to many existing agents has already been profiled and made publically available.

**Figure 1:**
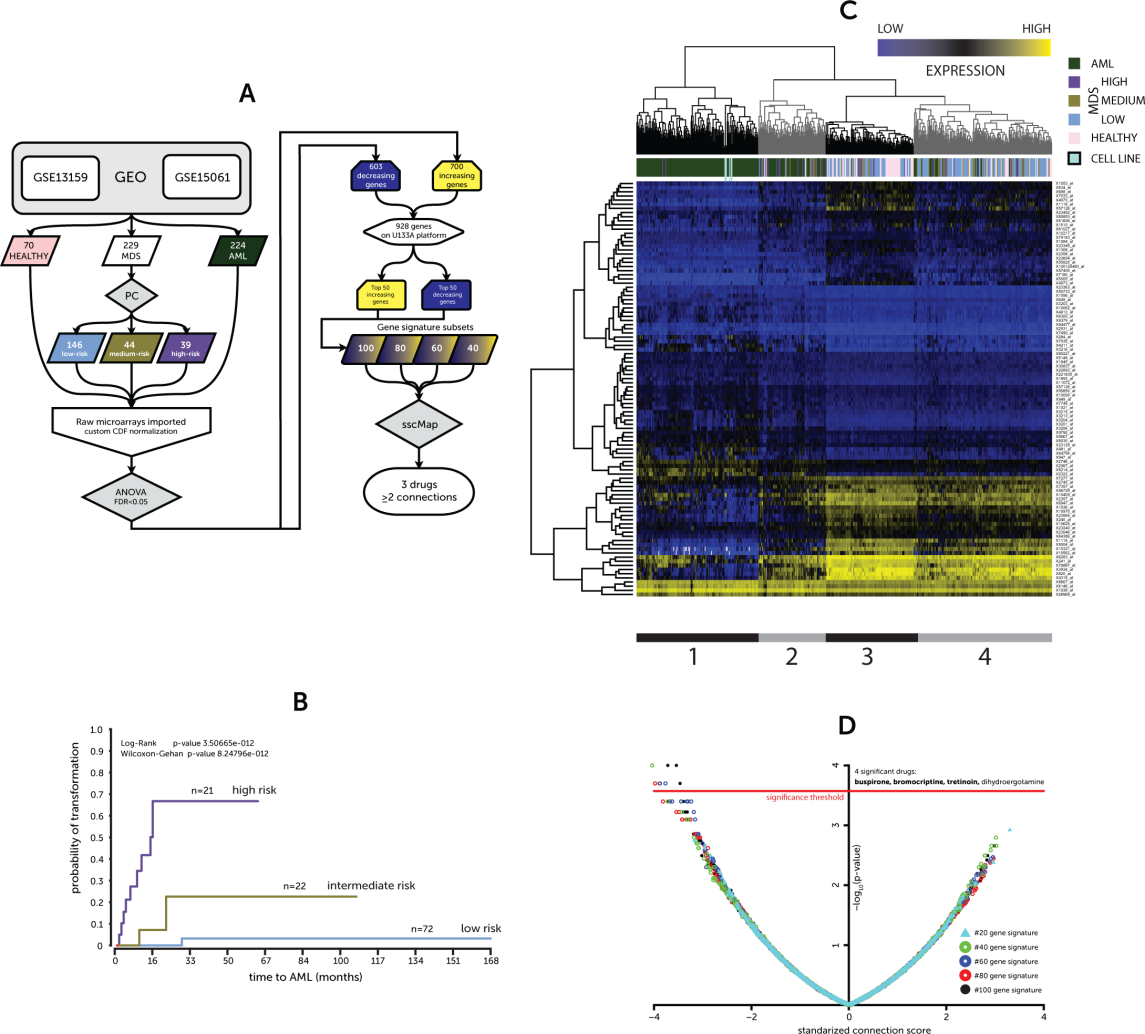
(**A**) Overview of analytical approach (**B**) Kaplan-Meier for probability of AML transformation (**C**) Heatmap of expression for 50–50 gene signature (**D**) sscMap drug-signature connections. (A) Flowchart of analysis pipeline used to generate gene signature and identify compounds using sscMap (B) A Kaplan-Meier plot illustrating the stratification of a subset of MDS patient gene expression profiles between the prognostic classes and their risk of leukemic transformation (low, medium & high risk of transformation) (C) Hierarchical cluster of patient and donor gene expression profiles based on the identified 50–50 gene signature. Colored bars indicate patient or donor status. The model cell lines included in the screen are also represented in the heatmap and are connected to the heatmap to highlight their positioning. The expression values are presented as absolute microarray intensity values with yellow representing high expression and blue low expression. The grey and black bars underneath delineate the four clusters in the dendrogram, which are further discussed in the text. (D) A volcano plot of the connection scores and *p-*values generated by the sscMap tool between the gene expression signature and the broad institute perturbagen library. Each point represents a derived gene signature to perturbagen/cell line connection. The red line represents the threshold of statistical significance (∼3.573, *p-*value = ∼2.675 × 10^−4^) for the sscMap algorithm. There were 8 significant connections across all the signatures, all of which had a negative score and represented 4 drugs. The drugs with ≥ 2 connections are in bold.

This research has applied a statistically significant variant of cMAP, sscMap, [9] to the task of identifying new therapies for MDS and AML (Figure 1A). This approach has revealed a novel potential therapy in re-purposing an existing and widely-prescribed drug, bromocriptine. The drug has shown selectivity for the leukaemic cell as well as synergy with current therapies.

## RESULTS

### Identification of gene signature of MDS development and progression

The gene expression dataset employed in this study was derived from two published datasets listed on the GEO repository as GSE13159 [10] and GSE15061 [11]. The derived sample set included healthy donors (70) and patients with MDS (229) or AML (224, including all risk groups). However, all those AML patients that had cytogenetic rearrangements that typically preclude prior undetected MDS were excluded from analysis, such as t (8;21) or inv (16). Mills, et al. previously developed a prognostic classifier (PC) that would stratify MDS patients by time to AML transformation based on their gene expression profiles [11]. The MDS sample profiles were classified using the PC as either, low (*n =* 146), medium (*n =* 44) or high risk (*n =* 39) of transformation (PC-Risk). Out of the 229 MDS gene expression profiles our group had access to linked survival and progression to AML times for 115. This subset of samples showed a similar Kaplan-Meier pattern between the risk group assigned by the PC and time to AML transformation (Figure 1B) to that shown in the original publication [11].

The gene expression profiles were imported as raw CEL files and normalized using the appropriate Brainarray Chip Definition File (CDF) providing a more concise and accurate group of probe sets based on up-to-date genome information [12]. Statistical analysis by ANOVA of the microarray data across the groups (normal, low, medium, high PC-Risk & AML) yielded a large list of differentially expressed genes (DEGs) meeting an FDR-adjusted *p-*value threshold of 0.05. Of these DEGs, 1, 303 showed a positive (700) or negative (603) fold-change for every pairwise comparison, i.e. consistently increasing or decreasing, comprising a signature of development and progression. However, only 928 DEGs have a probe set on all the U133A platforms used for connectivity mapping. Coincidentally, there were 464 increasing and 464 decreasing genes that could be used to interrogate the sscMap database. The top 50, ranked by *p-*value, of each of these lists (increasing and decreasing genes) formed the basis of a progression gene signature (50–50 gene list) (Supplementary Table 1).

In order to visualize the effectiveness of the 50–50 gene list in categorizing patients we then performed complete linkage clustering of both the samples and the changes in individual gene expression creating the heatmap seen in Figure 1C. Four clusters were identifiable; the leftmost black cluster (1) holds the majority of the AML samples and only a few high-risk MDS samples together with the model cell lines, representing a high-risk MDS (MDS-L) and AML (OCI/AML-3) in duplicate. The next cluster (2) is also predominated by AML samples and high-risk MDS samples. However, there are a few low-risk MDS and healthy samples. The third cluster (3) is predominated by healthy and low-risk MDS samples, including only a single AML sample. The final grey cluster (4) is more heterogeneous, but is also mostly healthy and low-risk MDS samples. Interestingly, the medium-risk MDS samples are distributed throughout all clusters, highlighting the heterogeneity of this group.

### Discovery of drug connections via sscMap

The top 50, 40, 30, 20 or 10 genes from the increasing and decreasing gene lists were combined to form 5 derivative gene signatures of 100, 80, 60, 40 and 20 genes; these 5 signatures were connected by sscMap to the Broad Institute cmap02 perturbagen database. Those compounds with a negative connection score (potential ‘inhibitors’ of development/progression) that were identified as significant by at least two signatures were considered of interest. Of the four drugs identified only 3 compounds (in bold) met the stricter criteria (Figure 1D). Importantly, all the significant connections in every comparison were for the leukemic cell line HL-60 in the cmap02 database. The compounds were buspirone, bromocriptine, and tretinoin (ATRA). Tretinoin (ATRA) has already proven successful in a number of clinical trials for MDS and AML [13, 14]. Its molecular action triggers greater cell differentiation and appears to lead to an increase in terminally-differentiated hematopoietic cells in patients when used in combination with other agents, such as EPO or epigenetic modifiers [15, 16]. The other two compounds belonging to the neuroleptic class of drugs, however, have never, to the knowledge of the authors, been trailed in the context of treating hematological malignancies.

### Screening of novel compounds

**Figure 2:**
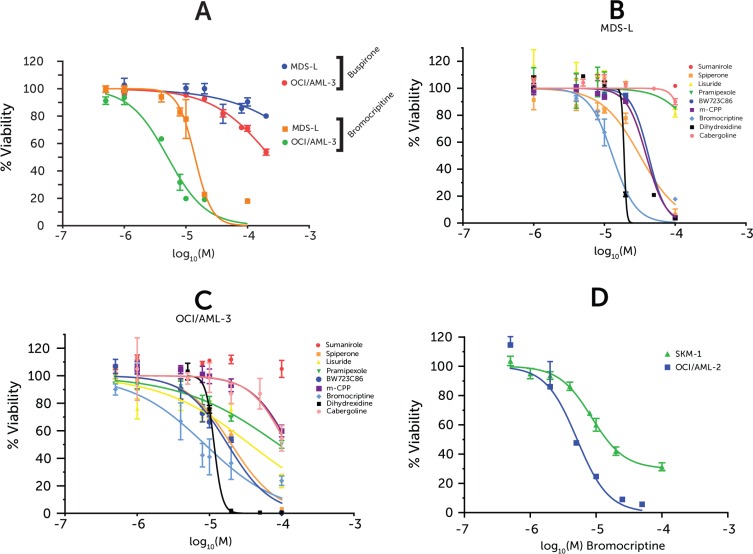
Drug dose-response in cell lines Cell viability measured via ATP-based high-throughput assay normalized to vehicle-treated control. Drug doses are represented as logarithm base 10 of Molarity. Points represent averages from 3 replicates and error bars represent SEM. (**A**) Bromocriptine & Buspirone screened against MDS-L and OCI/AML-3 (**B**) Neuroleptic compound screen against MDS-L (**C**) Neuroleptic compound screen against OCI/AML-3 (**D**) Bromocriptine-treated SKM-1 & OCI/AML-2 cell lines.

In light of the wealth of data surrounding tretinoin, only buspirone and bromocriptine were taken forward for initial screening in the MDS-L and OCI/AML-3 cells, representing high-risk MDS and AML, respectively. Bromocriptine, a dopamine agonist, was shown to be considerably toxic against both cell lines, and significantly more so than buspirone (Figure 2A). In order to assess whether bromocriptine's toxicity was common to other dopamine modulating agents, a panel of similar compounds were screened against the model cell lines, including some other neuroleptic class compounds (Figure 2B and 2C).

The expression of this neuroleptic class of receptors in the model cell lines was investigated in RMA-normalized microarray data. Only three (DRD2, DRD4, HTR3C) of the twenty possible dopamine / serotonin receptors demonstrated above background expression (> 4 log_2_ intensity value – red line) in all the cell line samples studied (Supplementary Figure 1A) — with even these exhibiting very modest (< 5.5 log_2_ intensity value – blue line) compared to overall probe set intensity distribution (Supplementary Figure 1B). This would suggest that it is unlikely that the cells are sensitive to signaling via the dopamine / serotonin receptors. The dopamine and serotonin family of receptors have many members and each compound tested showed different affinities for each. The panel included dopamine agonists against all 5 dopamine subtypes and those serotonin receptors for which bromocriptine has been shown to have affinity (K_i_ < 100 nM) [17] (Bold genes in Supplementary Figure 1A).

However, only one of the compounds tested elicited a viability response of similar magnitude as seen with bromocriptine: Dihydrexidine which is a dopamine 1 agonist, an activity which bromocriptine also possesses to some degree. However, it has an unfavorable pharmacokinetic profile, which limited its clinical utility, as it led to profound hypotension in the clinic [18]. Furthermore, it seems to have a very limited therapeutic window as its response profile has a much steeper curve. Two further model cell lines, SKM-1 and OCI/AML-2, were treated with bromocriptine eliciting a similar reduction in viability (Figure 2D).

### Bromocriptine induces apoptosis and decreases colony formation

**Figure 3:**
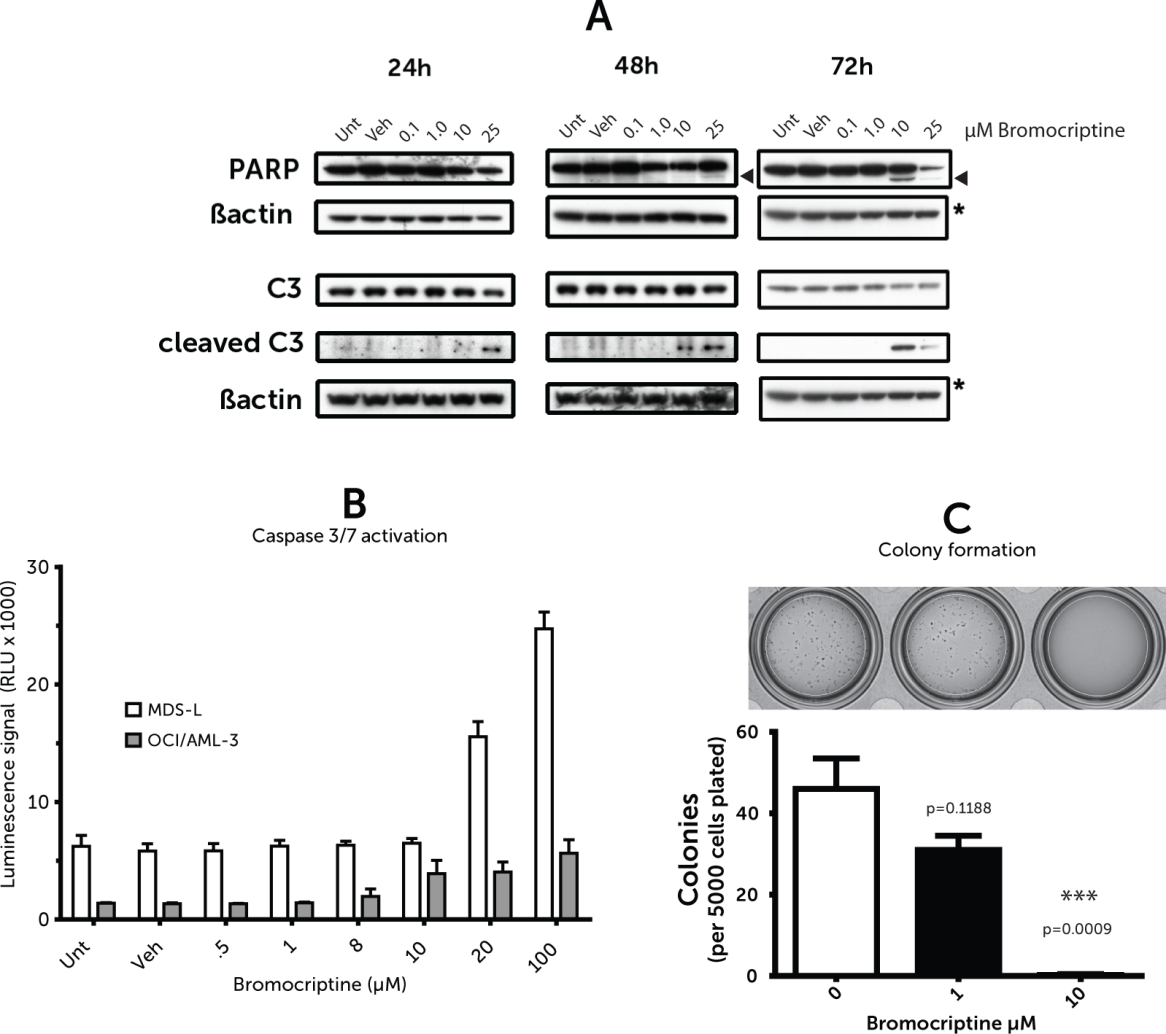
(**A**) Caspase/PARP cleavage (**B**) Caspase activity assay. (**C**) Methylcellulose colony assay. (A) Western blots of cell lysates probed for Caspase 3 (C3) and poly ADP ribose polymerase (PARP) and their cleaved forms at the labelled time points post bromocriptine treatment of OCI/AML-3 cells. Images are representative of westerns replicated three times. Arrowheads indicate cleaved PARP (B) Caspase 3/7 activity 24 h after bromocriptine treatment; measured via luminescent DEVD peptide-cleavage assay. Values represent averages from duplicates and error bars represent standard error of the mean. (C) Methylcellulose-based colony-forming assay for MDS-L cell line with representative image.

Cleavage of Poly ADP Ribose Phoshorylase (PARP), a commonly-employed marker of the final stages of apoptosis, was used to identify the mode of cell death elicited by bromocriptine. While PARP cleavage at 48 h was marginal, by 72 h it was substantial, following treatment with bromocriptine (Figure 3A) with doses close to the 72 h IC_50_ as estimated during the initial screen. Caspase 3 is upstream of PARP in the apoptotic cascade and is one of the enzymes that catalyze its cleavage. This protein is itself cleaved relatively early in apoptosis signaling; as seen in Figure 3A; there is demonstrable Caspase 3 cleavage by 24 h prior to any detectable PARP cleavage. Caspase 3 cleavage was also seen using a substrate-based assay (Figure 3B) and was seen equally in both the MDS-L and OCI/AML-3 cell lines.

To better predict *in vivo* effectiveness and investigate whether bromocriptine may have any effects on colony forming ability, methylcellulose assays were performed. MDS-L cells were pre-treated for 18 h with bromocriptine at various doses (0, 1, 10 μM) and seeded into cytokine supplemented methylcellulose. Equal cell numbers were used at the time of plating to correct for any cell death in the first 18h to specifically select for defects colony in formation. There was a very significant decrease in colony formation following bromocriptine treatment at 10 μM (Figure 3C).

### Synergy with standard agents and selectivity for the leukemic cell

**Figure 4:**
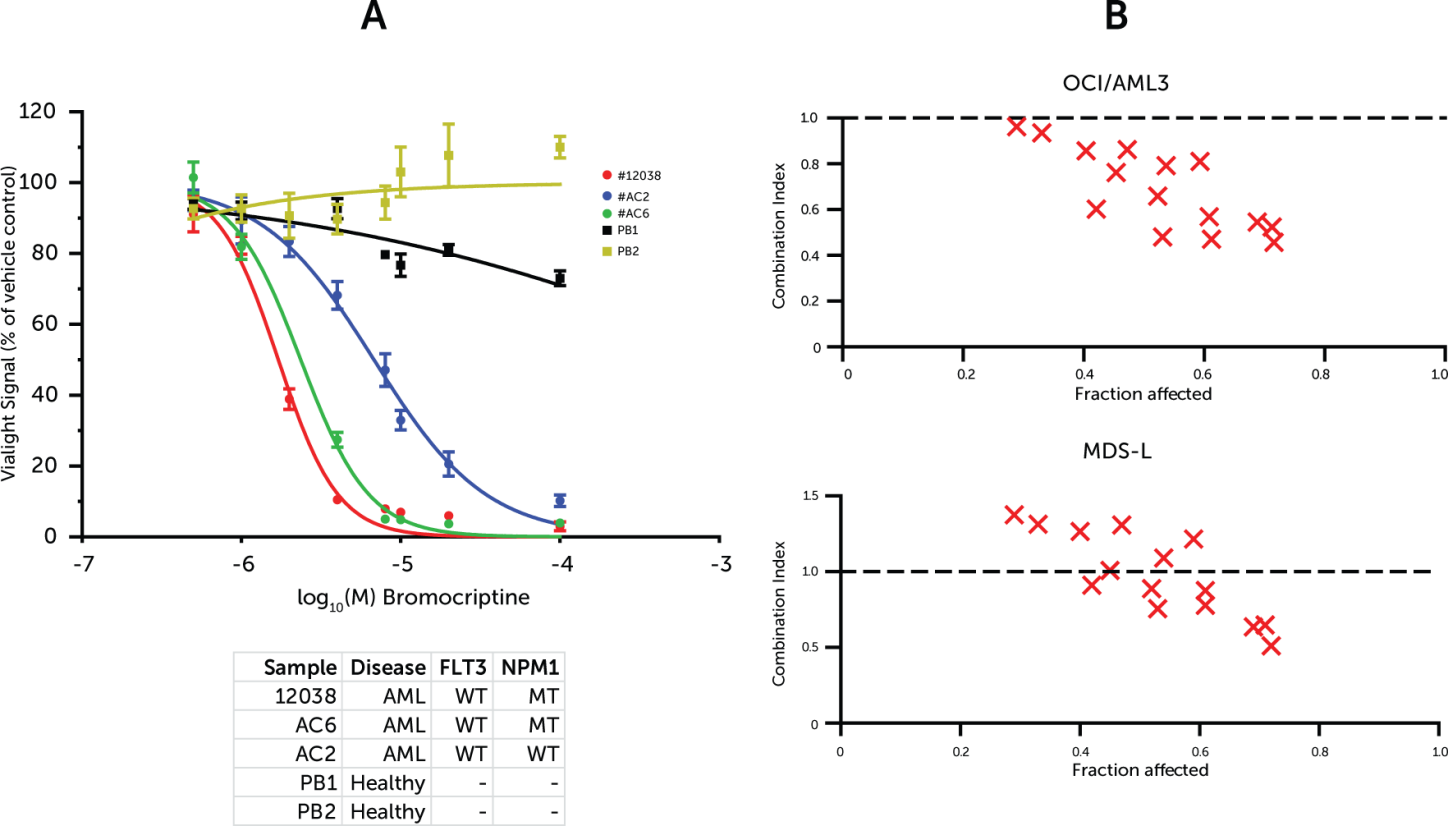
(**A**) Primary mononuclear cell response to bromocriptine (**B**) Synergy of Bromocriptine and cytarabine. (A) Dose-response curve using ATP-based viability assay normalized to vehicle-treated cells. Samples represent peripheral blood mononuclear cells (PBMCs) from diagnostic AML samples or mobilized bone marrow donors. Points represent averages from 3 replicates and error bars represent SEM. (B) CalcuSyn synergy plots illustrating Combination Indices for doses of cytarabine and bromocriptine at and around the individual drug 72 h IC_50_ values. Values at 1.0 represent additive effect, whereas values below 1.0 suggest synergistic effect.

The identification of bromocriptine via sscMap implied that it interferes with the specific transcriptional programs involved in MDS development and progression however its actual selectivity is not assured. To address this issue, mobilized peripheral blood mononuclear cells from two healthy donors, who had been conditioned using G-CSF together with three primary diagnostic AML samples were collected and their individual sensitivity to bromocriptine assessed. The results demonstrate a therapeutic window between the leukaemic and the healthy cells (Figure 4A). This infers that the action of bromocriptine is selective for the leukemic cell.

In order to assess the interaction of bromocriptine with routine leukemia therapeutics, synergy was evaluated by treating cells with combinations of bromocriptine and cytarabine. The cells were treated in liquid culture at 0, 0.25, 0.5, 1 and 2 × the 72H IC_50_ doses of each drug combinatorially, i.e. 25 (5 × 5) individual treatments (Figure 4B and Supplementary Figure 2). The Combination Index values were calculated using the CalcuSyn software according to the method previously reported by Chou-Talalay et al [19]. The OCI/AML-3 cell line exhibited synergism (CI < 1) at all doses tested. The MDS-L cell line showed synergism at the higher doses, with lower doses eliciting an additive effect (CI ≈ 1) and still lower doses implying mild antagonism (CI > 1). However, this low-dose antagonism is likely a limitation of the algorithm itself and is a common finding for anti-cancer drugs [20, 21].

## DISCUSSION

Several studies have reported alterations in the transcriptional program of cells in MDS patients both in relation to healthy cells and across severity of disease [22, 23]. This study has sought to harness these changes during the development and progression of the disease to identify potential novel therapeutic agents. This approach has uncovered a gene signature which showed a consistent increasing or decreasing trend in expression across the disease spectrum from normal bone marrow, MDS patients with low, intermediate and high risk of transformation and *de novo* AML. Clustering of the patient transcriptomes by these genes led to a number of distinct clusters, chiefly around the expected groups, reinforcing the suitability of the gene signature. Interestingly, the medium-risk MDS samples are distributed throughout all clusters. This perhaps reflects the clinical heterogeneity in those MDS patients that are neither indolent nor AML-like.

Perhaps not surprisingly, the gene signature itself contains many genes with established roles in MDS and AML. For example, 7 members of the HOX genes, a gene family known to be important in both normal and malignant hematopoiesis, are included [24]. Moreover, WT1, CDK6 and FLT3, well-established drivers of leukemogenesis, [25–27] also form part of the increasing component. The presence of these genes in the signature may indicate value in its investigation as a mechanism of monitoring disease progression. However, the principal goal of this research was to leverage this signature to identify new and re-purposed therapies for MDS using sscMap, without the need to screen a multitude of individual targets. The other benefit of the connectivity mapping approach is that re-purposed drugs are three times more likely to be approved than novel agents [5, 6].

The potential of connectivity mapping has been shown many times before [28–32], but we believe that this is its first reported use in identifying drugs to re-purpose for the treatment of MDS and AML. The use of the improved statistically-significant approach together with multiple gene signatures [29] and the updated, and more robust, chip definition file (CDF) microarray probe-mapping [12] had increased the chances of a successful hit. Indeed, the identification of ATRA by the sscMap algorithm added significant authority to and validation of the process. sscMap identified two other compounds with potential anti-leukemic effects, with bromocriptine proving itself worthy of further *in vivo* and clinical investigation given our positive pre-clinical results.

In terms of drug re-purposing, a compound which shows an additive or synergistic effect with established therapies is more likely to be accepted by both physicians and patients themselves. The current gold-standard in the treatment of high-risk MDS and AML is cytarabine. Of particular relevance is the apparent success of low doses of this compound in treating elderly MDS/AML patients unable to undergo more aggressive regimens [33–35]. In these cases any compound that enhances effectiveness without significant side-effect would be of huge benefit to these patients.

Bromocriptine shows synergy with cytarabine in the more proliferative OCI/AML-3 cell line at all doses tested. Its effect on the MDS-L cell line is less dramatic, but many doses do show synergy and the rest are mostly additive in their effect. This suggests that bromocriptine is more active in combination with cytarabine on more proliferative cells; selectivity that most chemotherapies aim for.

Bromocriptine as a therapeutic agent is non-toxic and has been widely-prescribed for decades with minimal side-effects. It is traditionally prescribed to treat pituitary-related syndromes, such as hyperprolactinemia, but it has also more recently found use in the management of Parkinson's symptoms. While at the highest dose used in this study it could be associated with increased side-effects in patients, these should be manageable in a combination setting, especially when compared to highly-toxic chemotherapy-only regimens. We have now shown it to possess efficacy against both leukemic and myelodysplastic cell lines. The exact mode-of-action of bromocriptine in achieving this remains unknown. Given the ineffectiveness of a host of other pharmacologically similar compounds in the same assay; its activity appears to be unrelated to its main action as a dopamine agonist. For the same reason, neither does it appear to concern its weaker engagement of other targets, such as serotonin receptors or excitatory amino acid transporters. These findings tally well with the lack of significant expression of this class of receptors on the model cell lines, in contrast to many solid tumours, [36] some of which show a proliferative response [37]. Furthermore, the compounds tested included Cabergoline, which is now superseding bromocriptine in the clinic [38], both of which are hypothesized to inhibit prolactin production [39]. The ineffectiveness of Cabergoline in our screens suggests it is unlikely that the prolactin inhibitory effect of bromocriptine explains it value. This suggests that bromocriptine has an as yet unknown molecular effect that triggers apoptosis in leukaemic cells. This is not the only case of a dopamine modulator demonstrating an anti-cancer effect, suggesting that this class of compounds is worth further investigation [40]. Indeed, there is a case study dating from 1981 of a diabetic patient on chronic bromocriptine therapy demonstrating marked leukopenia [41]. Interestingly, this unexplored territory may explain the growing correlative evidence that patients with certain unrelated diseases, including Parkinson's, for which bromocriptine is a therapy, have lower than expected rates of certain cancers [42].

Beyond its anti-proliferative and cytotoxic effects, bromocriptine demonstrates a preference for proliferative leukemic cells and appears to alter colony formation. These are key qualities in any hopeful treatment for bone marrow disease. All of these virtues, combined with its synergy with the gold-standard therapy make it a very exciting drug. It has the potential to improve the quality of life and longer-term outcome for those MDS and AML patients for whom high-intensity chemotherapy regimens are not an option.

## MATERIALS AND METHODS

### Gene expression datasets

The gene expression dataset employed in this study was derived from two published datasets listed on the GEO repository as GSE13159 [10] and GSE15061 [11]. Both contain Affymetrix array data from mononuclear cell fraction transcriptomes. GSE13159 contains a mixture of both bone marrow and peripheral blood samples, whereas GSE15061 contains exclusively bone marrow. Only those profiles from normal or complex karyotype AML, MDS or healthy donor samples were included in this study. The OCI/AML-3 and MDS-L cell lines were also profiled in-house on the Affymetrix platform using the same protocol and arrays as that published in the original MILE study mentioned above (GSE13159).

### Prognostic classification stratification

The prognostic classifier (PC) algorithm previously published and described by Mills et al [11] was developed from a data set comprising samples from healthy donors, a range of MDS subtypes and AML. This classifier was robustly shown to be able to stratify patients into one of 3 groups (low, medium and high) based on their risk of transformation to AML. The MDS gene expression profiles from the combined datasets of 229 samples used in this study were stratified using this algorithm to classify each raw CEL file. This stratification was subsequently incorporated in further statistical analyses (as PC-Risk).

### Identification of gene signature

The 229 original CEL files were downloaded and decompressed from the GEO online repository. The raw CEL files were imported into Partek Genomics Suite (PGS, version 6, St. Louis, Missouri 63005 U.S.A.). The data were normalized using RMA background correction with probe GC-content correction, quantile-normalization and median polish probe set summarization. During the import procedure the HGU133Plus2 Brainarray Chip Definition File (ENTREZG CDF version 16) was used to provide a more concise and accurate group of probe sets based on up-to-date probe sequence to NCBI Entrez Gene alignments [12]. The ANOVA algorithm in PGS was used to generate *p-*values for each ENTREZ-based probe set to identify significantly varying gene expression across the 5 groups (normal, low/medium/high PC-Risk & AML). The following comparisons were also included as separate one-way ANOVAs to generate fold changes via least-squares means of each group (LS-means). Any *p-*values generated were moderated using Benjamini-Hochberg false discovery rate (FDR) [43].

*Normal* vs *Low PC-Risk MDS*
*Low PC-Risk MDS* vs *Medium PC-Risk MDS*
*Medium PC-Risk MDS* vs *High PC-Risk MDS*
*High PC-Risk MDS* vs *AML*

### sscMap identification of perturbagens

The sscMap algorithm depends on a reference set of gene expression profiles that represent various perturbagen-treated cells. The Broad Institute cmap02 profiles were used in this study. They consist of 3730 individual reference profiles from a panel of ∼1000 compounds applied at various doses to the PC3, MCF7 and HL-60 cell lines. The raw CEL files were downloaded and normalized using the Affymetrix-power-tools RMA-sketch algorithm and the platform-appropriate Brainarray ENTREZG v16 CDF file, similarly to the study dataset. The reference data were from 3 separate platforms and therefore normalized in 3 batches. During the build of the “refset”, required by the sscMap tool, only those probe sets common to all platforms were included.

The previously identified gene signatures were formatted into tab-delimited tables of Probe IDs and positive or negative integers representing their ranks and up or down-regulation. These tables were then loaded into the sscMap tool. The algorithm was run, whereby it additionally generated 10, 000 random signatures of length corresponding to each signature and compared each with the Broad Institute “refset”. The tool then returned a list of positive or negative connection scores for each compound against each signature and a *p-*value based on the random permutation test. The *p-*value significance cut-off was based on an accepted false-positive rate of 1 per analysis, in other words 1/N where *N =* the number of comparisons, in this case 3730 reference profiles therefore ∼0.00027. This *p-*value in the form −log_10_ (*p-*value) is equivalent to 3.572.

### Cell lines and primary material

All cell lines were maintained at 37°C and 5% CO_2_ and cultured in RPMI1640+NaHCO_3_+L-Glut (Sigma-Aldrich Company Ltd., Dorset, UK) supplemented with at least 10% FBS Superior (Biochrom AG) and 1% Penicillin/Streptomycin. The OCI/AML-3 and OCI/AML-2 cell lines were sourced from DSMZ. The MDS-L cell line was a kind gift from Taoru Kohyama at the Kawasaki Medical School, Japan [44]. Its media was further supplemented with 1 mM UltraGlutamine I (Lonza Cologne GmbH, Köln, Germany), 10 mM HEPES (Cell Culture Grade, Sigma-Aldrich), 20 ng/mL hGM-CSF (Cell Guidance Systems Ltd., Cambridge, UK) and 10 IU/mL hIL-3 (CellGenix GmbH, Freiburg, Germany). The SKM-1 cell line was a kind gift from Stefan Fröhling (Nationales Centrum für Tumorerkrankungen, Heidelberg, Germany). Both the SKM-1 and MDS-L cell lines were validated using STR analysis as unique and pure (LGC Standards, Middlesex, UK). The SKM-1 STR markers matched those recorded in the DSMZ library. The MDS-L cell line, as it is not available from cell banks, was shown by karyotyping to have monosomy 7 and del (5q) as cited in the originating paper (Supplementary Figure 3) [44].

Ficolled bone marrow or peripheral blood samples from AML patients, obtained with consent, at diagnosis (Northern Ireland Ethical Approval: 08/NIR01/9) were diluted one in two with phosphate-buffered saline and then layered over one-third volume of Ficoll Paque Plus (GE Healthcare Life Sciences, Buckinghamshire, UK). After 30 minutes of centrifugation at 400 × *g* the buffy coat containing mononuclear cells was removed and washed twice with RPMI1640. Cells were then counted and cryogenically frozen in freezing medium (10% DMSO, 50% RPMI1640, 40% FBS). When required, cells were thawed at 37°C, washed once with RPMI-1640 and re-suspended in 10% FBS in RPMI1640 before treatment.

### Screening compounds

Bromocriptine and Busprione were purchased from Tocris Bioscience (Bristol, UK) as mesylate and hydrochloride salts respectively.

### Other dopamine and serotonin agonists

All other screening compounds, except m-CPP (Sigma-Aldrich) were from Tocris Bioscience: Sumanirole (D_2_ agonist), Spiperone (5-HT_2A_ serotonin and selective D_2_-like dopamine receptor antagonist), Lisuride (D_2∼4_ agonist), Pramipexole (D_2∼4_ agonist), BW723C86 (5-HT_2B_ receptor agonist), m-CPP (Pan-serotonin agonist), Dihydrexidine (full agonist at the dopamine D1 and D5 receptors), Cabergoline (D_2∼4_ agonist, possible prolactin inhibitor), DL-TBOA (EAAT blocker)

### Viability assay and synergy calculation

Cell viability post drug treatment was measured using the ViaLight^™^ ATP-based assay (Lonza), typically at 72 hours, unless otherwise stated. Relative Luminescent Units were normalized as a percentage of time-matched vehicle-treated control samples. Synergy was assessed by treating cells with the various combinations of 0.25, 0.5, 1 and 2 × the 72H IC_50_ doses of both bromocriptine and cytarabine. The Combination Index values were calculated using the CalcuSyn software according to the method of Chou-Talalay et al [19, 45].

### Western blotting

Cell lysates post-treatment were prepared using ProteoJET Cell Lysis Reagent (Fermentas, Vilnius, Lithuania) supplemented with Pierce Protease Inhibitor Cocktail (Thermo Scientific, Hemel Hempstead, UK) and 10 mM Sodium Fluoride and 20 mM Sodium Orthovanadate (Sigma-Aldrich). Lysates were then denatured at 70°C for 10 minutes in Bolt^™^ LDS-based loading buffer supplemented with DTT (Life Technologies, Paisley, UK). The denatured samples were run on Bolt^™^ 12% Bis-Tris gels (Life Technologies) and transferred using standard Towbin Western transfer to 0.2 μm Immobilon PVDF membranes (Millipore, Watford, UK). Antibodies were used at 1:1000: Cleaved Caspase 3 (Cell Signaling Technology, Leiden, Netherlands), Caspase 3 (CST), PARP (C-2–10, Enzo Life Sciences, Exeter, UK) & β-actin (A5441, Sigma-Aldrich). Appropriate secondary antibodies (Dako, Ely, UK) were used at half the primary antibody concentration and developed using WesternBright ECL Reagent (Advansta, Menlo Park, USA).

### *Ex vivo* methylcellulose

Cells were treated for 18 hours in liquid culture and counted. Subsequently 2 × 10^4^ cells per mL were thoroughly mixed with pre-supplemented Methylcellulose media, without drug (HSC003, R & D Systems) and seeded into a 12-well plate at 250 μL per well. After 9 days colonies were stained with 25 μL of 8 mg/mL Iodonitrotetrazolium (Sigma-Aldrich) solution in pure ethanol. After overnight staining, colonies were imaged and counted using the GelCount system (Oxford Optronix, Abingdon, UK) using consistent settings for all plates.

## SUPPLEMENTARY MATERIALS TABLES AND FIGURES




