# Supplementary material for: Identification and validation of the dopamine agonist bromocriptine as a novel therapy for high-risk myelodysplastic syndromes and secondary acute myeloid leukemia
Source: Oncotarget. 2015 Dec 28;7(6):6609–19. doi: 10.18632/oncotarget.6773 (PMC4872736; doi:10.18632/oncotarget.6773)
Supplement: Supplementary file 1 [file oncotarget-07-6609-s001.pdf]

Identification and validation of the dopamine agonist bromocriptine as a novel therapy for high-risk myelodysplastic syndromes and secondary acute myeloid leukemia

Supplementary Materials

Supplementary Table 1: List of genes in 50-50 signature used in sscMap analysis

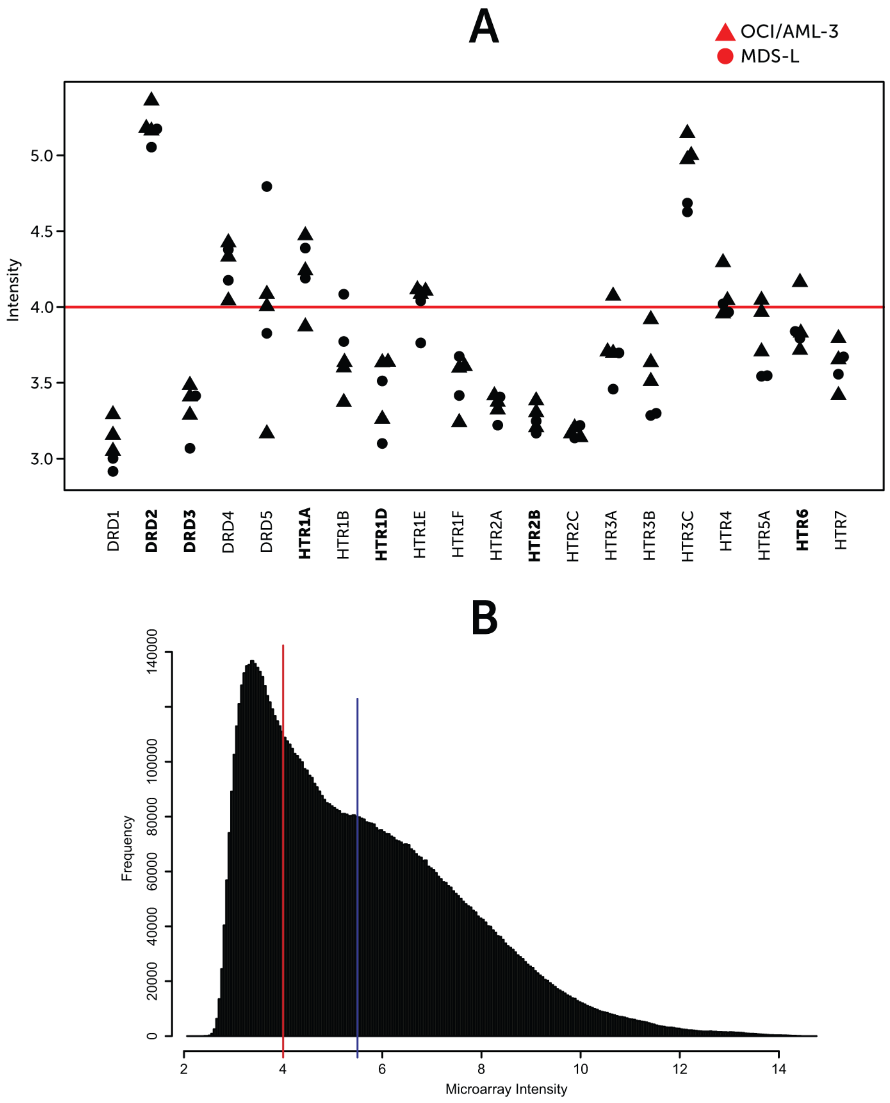

Supplementary Figure S1: (A) Expression of dopamine and serotonin receptors on MDS-L and OCI/AML-3 cell lines. *Y*-axis represents normalized microarray intensity value. Bold gene labels indicate receptors for which bromocriptine has been shown to have affinity ($K_i < 100$ nM). Red-line indicates detection threshold for expression. (**B**) Histogram of normalized microarray intensity values for all samples included in the analysis. Red line signifies detection threshold for expression, as above. Blue line indicates threshold for low-expression genes.

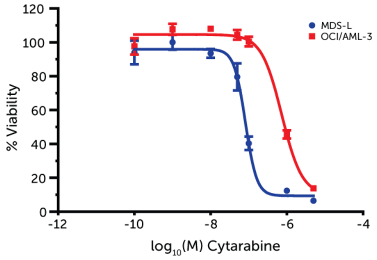

× Bromocriptine IC_{50} | × Cytarabine IC_{50}

	0	0.25	0.5	1	2
**0**	0 | 0	0 | 0.25	0 | 0.5	0 | 1	0 | 2
**0.25**	0.25 | 0	0.25 | 0.25	0.25 | 0.5	0.25 | 1	0.25 | 2
**0.5**	0.5 | 0	0.5 | 0.25	0.5 | 0.5	0.5 | 1	0.5 | 2
**1**	1 | 0	1 | 0.25	1 | 0.5	1 | 1	1 | 2
**2**	2 | 0	2 | 0.25	2 | 0.5	2 | 1	2 | 2

IC_{50}	Bromocriptine	Cytarabine
**OCI/AML-3**	6μM	736nM
**MDS-L**	11μM	82nM

Supplementary Figure S2: Dose-response curves for MDS-L and OCI/AML-3 cell lines treated with cytarabine. Cell viability measured via ATP-based high-throughput assay normalized to vehicle-treated control. Drug doses are represented as logarithm base 10 of Molarity. Points represent averages from 3 replicates and error bars represent SEM. Tables illustrate treatment regime for synergy assay and IC_{50} doses for MDS-L and OCI/AML-3 with bromocriptine and cytarabine.

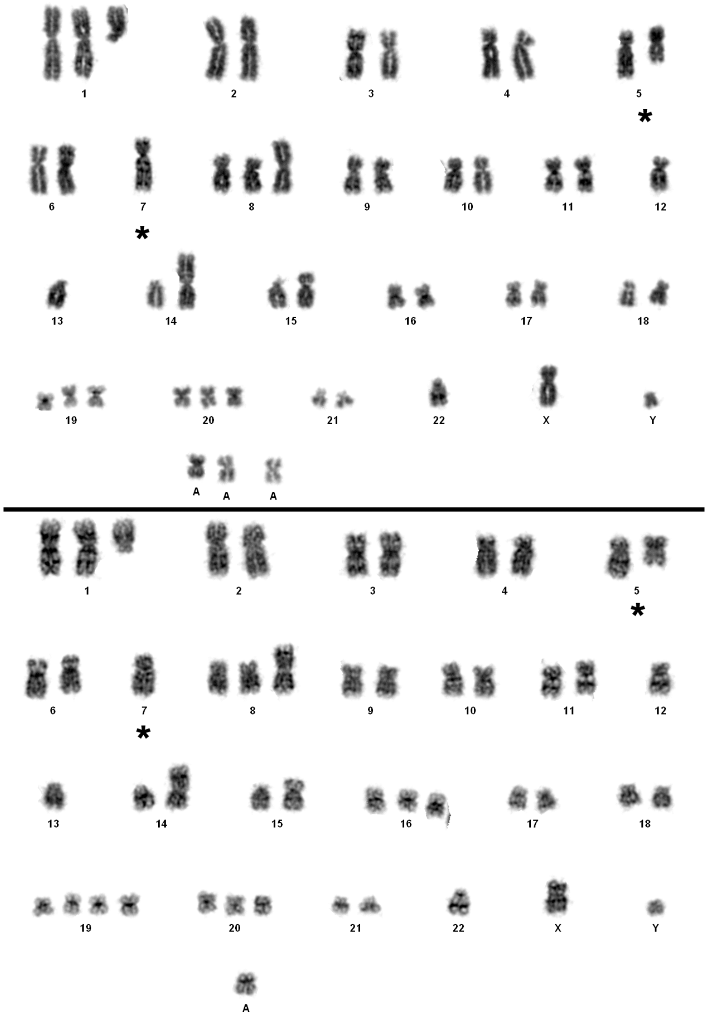

Supplementary Figure S3: Two representative metaphase spreads from MDS-L cell line showing characteristic del(5q) and monosomy 7 (indicated by asterisks).
